# Deutschlands erste roboterassistierte postmortale Nierentransplantation: eine gute Option bei Adipositas permagna – wenn das Team stimmt

**DOI:** 10.1007/s00120-024-02459-4

**Published:** 2024-10-09

**Authors:** Philipp Reimold, Christer Groeben, Christian Keil, Fabian Kormann, Liliane Peters, Christian Volberg, Birgit Kortus-Götze, Johannes Wild, Joachim Hoyer, Luka Flegar, Johannes Huber

**Affiliations:** 1 Klinik für Urologie, Philipps-Universität Marburg, Baldingerstraße, 35043 Marburg, Deutschland; 2 Klinik für Anästhesie und Intensivtherapie, Philipps-Universität Marburg, Marburg, Deutschland; 3 Klinik für Innere Medizin, Nephrologie und Internistische Intensivmedizin, Philipps-Universität Marburg, Marburg, Deutschland

**Keywords:** Nierentransplantation, Roboterassistierte Chirurgie, Postmortale Spenderniere, Adipositas, Fallbericht, Kidney transplantation, Robot-assisted surgery, Deceased donor kidney, Obesity, Case report

## Abstract

Roboterassistierte Nierentransplantationen werden in Europa seit 2015 durchgeführt. In Deutschland verbreitet sich die Technik langsam – zuletzt führten 3 urologische Zentren diese Art der Eingriffe durch. Im Folgenden präsentieren wir den ersten Fall einer postmortalen, roboterassistierten Nierentransplantation in Deutschland bei einem 60-jährigen Patienten. Neben der OP-Technik und dem klinischen Verlauf wird insbesondere auf Chancen dieser OP-Technik und auch deren Herausforderungen eingegangen. Zusammenfassend deckt sich die Erfahrung an unserem Zentrum mit den Erkenntnissen aus der Literatur, dass die roboterassistierte postmortale Nierentransplantation eine sichere Methode ist, um die Prinzipien der offenen Transplantationschirurgie um die Vorteile der minimal-invasiven Methodik zu ergänzen.

**Video online:**

Die Online-Version dieses Artikels (10.1007/s00120-024-02459-4) enthält ein Video der Operation.

## Abstract (en)

Robot-assisted kidney transplantations have been performed in Europe since 2015. In Germany, the technique is slowly spreading - most recently, 3 urological centers performed this type of procedure. We present the first case of a post-mortem, robot-assisted kidney transplantation in Germany in a 60-year-old patient. In addition to the surgical technique and the clinical course, the opportunities of this surgical technique and its challenges are discussed in particular. In summary, the experience at our center is consistent with the findings from the literature that robot-assisted post-mortem kidney transplantation is a safe method for supplementing the principles of open transplantation surgery with the advantages of minimally invasive methods.

## Anamnese

Wir erhielten das Angebot einer postmortalen Spenderniere für einen 60-jährigen, adipösen Patienten (Body Mass Index [BMI] 39 kg/m^2^). Dieser wurde aufgrund einer chronischen Glomerulonephritis bei angeborener Einzelniere seit 14 Jahren mit Hämodialyse behandelt. Wenige Wochen zuvor war ein Organangebot an unserem Zentrum aufgrund der Adipositas und laufender Clopidogrel-Therapie abgelehnt worden.

## Befund

Bei Ankunft in unserer Klinik bot der Patient ein Serumkreatinin von 7,9 mg/dl bei kompensierten Elektrolyten. Die Diureseleistung betrug ca. 1500 ml/24 h. Präoperativ erfolgte eine Hämodialyse unter regionaler Antikoagulation mit Citrat. Die Thrombozytenaggregationshemmung hatten wir im Vorfeld auf ASS umgestellt.

## Diagnose

Neben der terminalen Niereninsuffizienz lagen eine koronare Zwei-Gefäß-Erkrankung mit Zustand nach Angioplastie und 3-facher Stenteinlage sowie eine paroxysmale supraventrikuläre Tachykardie mit Zustand nach interventioneller Ablation und eine ausgeheilte Hepatitis B vor. Des Weiteren bestand ein Nikotinabusus mit 25 „pack-years“.

Zur Evaluation der pelvinen Gefäßkalzifikationen bei den oben genannten Vorerkrankungen wurde ein Nativ-CT Abdomen durchgeführt. Hier zeigte sich im Bereich der Vasa iliaca externa ein Bereich ohne größere Kalzifikationen, sodass uns eine erfolgreiche Gefäßanastomose möglich erschien.

Das Spenderorgan (Niere rechts) erreichte unser Zentrum mit einer kalten Ischämiezeit von 7 h 20 min. Das Präparat wies eine ca. 2 cm distal des Aortenabgangs durchtrennte Nierenarterie auf, die kurze Nierenvene wurde mit einem Cavapatch im Rahmen der Backtable-Präparation verlängert. Das Missmatch betrug 2‑2‑1, der Spender war CMV-negativ, der Empfänger positiv. Als Todesursache des Spenders wurde eine Meningitis angegeben.

## Therapie und Verlauf

Aufgrund der Adipositas permagna entschieden wir uns zur roboterassistierten Nierentransplantation der rechten Spenderniere in die rechte Fossa iliaca. Die im Rahmen der Entnahme durchtrennte Nierenarterie bestärkte uns in dieser Entscheidung. Da uns das Spenderorgan an einem Sonntag erreichte, hatten wir im Vorfeld des Eingriffs das pflegerische und ärztliche Team unseres robotischen Transplantationsprogramms aus dem Dienstfrei aktiviert (siehe Abb. 4). Die robotische Technik zur Transplantation im Rahmen der Lebendspende hatten wir 12/2023 mithilfe der Kollegen aus Homburg/Saar implementiert, nachdem wir die robotischen Donornephrektomien seit 05/2023 erfolgreich zum Marburger Standard entwickelt hatten. Zum Eingriffszeitpunkt hatten wir im Rahmen von Lebendspenden 15 Donornephrektomien und 6 Nierentransplantationen erfolgreich roboterassistiert durchgeführt.

**Abb. 1:**
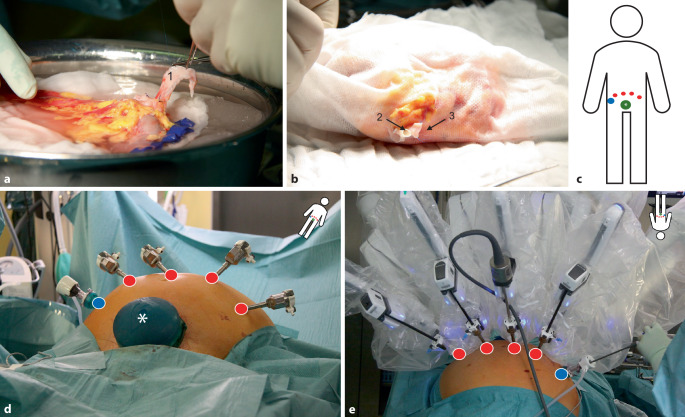
Backtable-Präparation des Spenderorgans im Eiswasser (**a**) inklusive durch Cava-Patch verlängerte Vene (*1*). Einwickeln des Transplantates in steriles Vlies (**b**) mit Exposition der präparierten Nierenarterie (*2*) und Nierenvene (*3*). Trokarplatzierung des robotischen Assistenzsystems bei 30° Trendelenburg-gelagertem Patienten (**c**–**e**). Es werden vier 8‑mm-Trokare (*rot*) und ein 12-mm-Trokar (*blau*) platziert. Zum Einbringen des Transplantats wird über einen Pfannenstielschnitt ein GelPort® (Applied Medical, CA, USA; *grün*, *Sternchen*) installiert

**Abb. 2:**
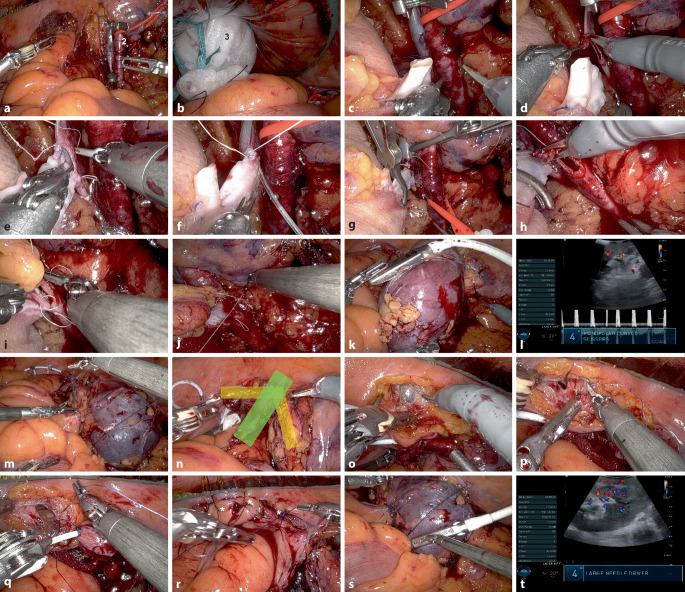
Operative Schlüsselstellen der robotisch assistierten Nierentransplantation: **a** Präparation der V. (*1*) und A. iliaca externa dextra (*2*). **b** Einbringen des Transplantats (*3*) durch den GelPort®. **c** Approximierung der Hilusgefäße an die Iliakalachse und **d** Venotomie. **e** Fortlaufende venöse Anastomose. **f** Ausspülen mit Heparin. **g** Freigabe der venösen Iliakalachse und Klemmen der Spendervene sowie der A. iliaca externa. **h** Arteriotomie und **i** arterielle Anastomose. **j** Freigabe der arteriellen Iliakalachse und Nachstich bei Blutung aus der arteriellen Anastomose. **k** Intraoperative Sonografie mit **l** gutem duplexsonografischem Perfusionsmuster. **m** Fixierung des Transplantats mittels Peritoneallappen. **n** Durchzug des Transplantatharnleiters (*gelb*) unter dem Ductus deferens (*grün*). **o** Inzision des Blasenperitoneums bis zur Blasenschleimhaut (*4*). **p** Eröffnung der Blasenschleimhaut und **q** Beginn mit der ureterovesikalen Anastomose . **r** Antirefluxplastik  mittels Einzelknopfnähten (*Sternchen*) des Blasenperitoneums. **s** Abschlusssonografie mit **t** gutem Perfusionssignal auch in der Peripherie

**Abb. 3:**
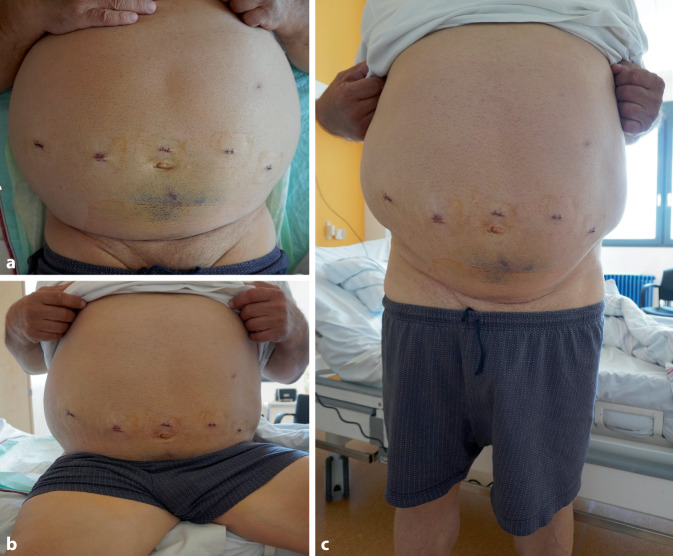
Postoperatives Ergebnis. Reizlose Narbenverhältnisse im Liegen (**a**), Sitzen (**b**) und Stehen (**c**) an POD 19. Das Abdomen verdeckt den Großteil der Pfannenstielinzision

Die Lagerung und Portplatzierung ist in Abb. 1 dargestellt und erfolgte nach unserem hausinternen Standard, einer Modifikation etablierter Techniken aus Homburg/Saar und Florenz [1, 2]. Die Konsolenzeit betrug 159 min, wobei 15 min auf die venöse, 21 min auf die arterielle und 20 min auf die ureterale Anastomose entfielen. Schlüsselmomente der Operation sind in Abb. 2a–t dargestellt und beschrieben. Eine Videozusammenfassung ist als E‑Supplement verfügbar und über obigen QR-Code abrufbar. Der eingebrachte DJ-Katheter zeigte sich intraoperativ okkludiert, sodass dieser anschließend transurethral entfernt und auf eine Harnleiterschienung verzichtet wurde. Der postoperative Verlauf gestaltete sich unauffällig, das Kreatinin des Patienten fiel auf 1,6 mg/dl am Entlasstag (POD 19), es war keine postoperative Dialyse notwendig. Postoperative Komplikationen traten nicht auf. Die Wundverhältnisse vor Entlassung zeigten sich schmerz- und reizlos und sind in Abb. 3 dargestellt. Auch 30 Tage postoperativ berichtete der Patient über keine Auffälligkeiten. Unter Immunsuppression mittels Ciclosporin A, Mycophenolat-Mofetil, Prednisolon und Basiliximab bot er ein stabiles Kreatinin von 1,6 mg/dl bei unauffälligem sonografischem Befund. 60 Tage nach Transplantation betrug die Ausscheidung ca. 2500 ml/24 h bei weiter stabilem Serumkreatinin von 1,47 mg/dl und einer endogenen Kreatinin-Clearance von 82 ml/min. Auch sonografisch zeigte sich die Niere weiterhin homogen perfundiert ohne Harntransportstörung oder Lymphozele, die Widerstandsindices lagen um 0,68.

**Abb. 4:**
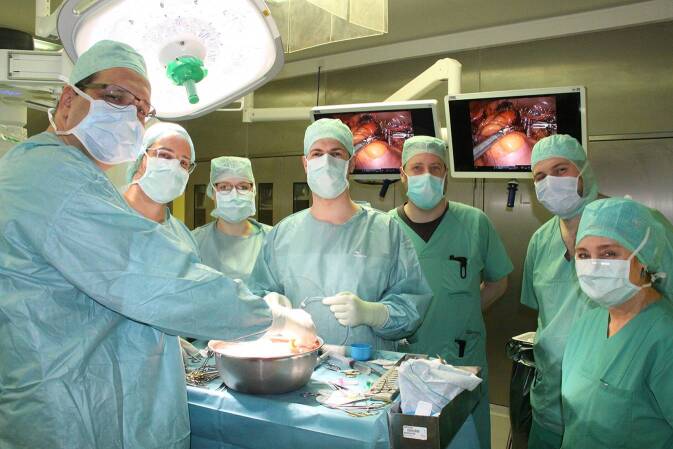
Das OP-Team bei der Backtable-Präparation (*v. l. n. r.*): J. Huber, C. Keil, L. Peters, P. Reimold, C. Volberg, F. Kormann, E. Bonnard

## Diskussion

Wir beschreiben die deutschlandweit erste roboterassistierte Nierentransplantation einer postmortalen Spenderniere. Die Technik ist an Zentren mit Erfahrung in der robotischen Lebendnierenspende und der offenen Transplantation sicher durchführbar [3] und bietet eine gute Alternative zur offenen Transplantation – insbesondere für Patienten mit Adipositas permagna [4, 5]. In Europa werden roboterassistierte Nierentransplantationen in 18 urologischen Zentren durchgeführt, darunter 2 weiteren in Deutschland (Homburg/Saar und Halle/Saale). Die Evaluation der Eingriffe erfolgt im Rahmen der gemeinsamen Datenbank der ERUS-RAKT-Gruppe, seitdem 2015 der erste Eingriff dieser Art in Europa durch Doumerc et al. durchgeführt wurde [6]. Die Chirurgischen Kliniken in Kiel und Heidelberg transplantieren ebenfalls robotisch, aber keines der 4 deutschen Zentren hat bislang postmortale roboterassistierte Transplantationen realisiert. Die Erfahrung an unserem Zentrum deckt sich mit den Erkenntnissen aus der Literatur, dass die roboterassistierte postmortale Nierentransplantation eine sichere Methode ist, um die Prinzipien der offenen Transplantationschirurgie um die Vorteile der minimal-invasiven Methodik zu ergänzen [7]. Erste Analysen aus dem ERUS-RAKT-Konsortium weisen auf eine Nicht-Unterlegenheit der roboterassistierten im Vergleich zur offenen Nierentransplantation hin [1]. Die Herausforderungen sind jedoch nicht nur rein technischer, sondern v. a. organisatorischer Natur. Die Verfügbarkeit eines robotischen Operationssystems auch außerhalb der regulären Arbeitszeiten setzt die Anwesenheit geschulter MitarbeiterInnen voraus – vom OP- und Anästhesiepflegepersonal über die OP-Assistenz bis hin zum Operateur kann der Ausfall eines Akteurs den erfolgreichen Eingriff verhindern. Auch im beschriebenen Fall kamen 2 Mitarbeiter außerhalb ihrer regulären Dienstzeit in die Klinik, um die Operation zu ermöglichen. Auch an die Verfügbarkeit von roboterspezifischem Sterilgut muss man bei ungeplanten Eingriffen denken. Um diese Voraussetzungen zu gewährleisten, sind sowohl ein etabliertes offenes Transplantationsprogramm in der Abteilung als auch extensive Erfahrung mit roboterassistierten Eingriffen notwendig. Die präoperative Planung und Risikostratifizierung sind extrem wichtig, um den Erfolg einer roboterassistierten Transplantation zu gewährleisten. Neben der oben erwähnten präoperativen Bildgebung zur Einschätzung vaskulärer Kalzifikationen ist auch eine wiederholte intraoperative Reevaluation der Situation notwendig. In unserem Fall führten wir vor dem Einbringen des Transplantats ein Probeclamping durch, um einen kompletten Gefäßverschluss durch die Endo-Bulldogklemmen zu sichern. Das Ausmaß des viszeralen Fettes bei adipösen Patienten bleibt nach wie vor eine Herausforderung. Möglicherweise bietet eine präoperative Bildgebung und Beurteilung relevanter Schlüsselstrukturen Hinweise für den Anteil an störendem viszeralem Fett im OP-Gebiet [8].

Die Ausbildung der nächsten Generation von robotisch transplantierenden Teams kann durch standardisierte Ausbildungsprogramme, z. B. innerhalb des ERUS-RAKT-Verbunds, unterstützt werden [9]. So könnte diese vielversprechende Technik in Zukunft auch in die Breite der Transplantationschirurgie gebracht und v. a. bei Patienten angewendet werden, die am meisten von den Vorteilen der minimal-invasiven Chirurgie profitieren. Für sehr schlanke PatientInnen oder bei engen Platzverhältnissen erscheint uns die offene Technik jedoch nach wie vor überlegen.

## Fazit für die Praxis

- Die roboterassistierte Nierentransplantation ist nicht nur bei Lebendspenden, sondern auch bei postmortalen Organspenden an Zentren mit entsprechender Erfahrung gut und sicher durchführbar.
- Sie ergänzt Vorteile der offenen Transplantationschirurgie um die Vorteile der roboterassistierten Chirurgie, welche in der Urologie schon lange etabliert ist.
- Somit ist diese Technik für alle Patienten, die aufgrund ihrer Adipositas von minimal-invasiven Methoden profitieren, eine gute Alternative zur offenen Transplantation.

## Supplementary Information

Das als Supplement bereitgestellte Video stellt eine Zusammenfassung mit Schlüsselschritten des Eingriffes dar

## Abkürzungen

BMI: Body Mass Index
DJ: Double-J-Katheter (Harnleiterschiene)
ERUS: European Robotic Urological Society
POD: Post-operative day
RAKT: Robot-assisted kidney transplantation

## References

[1] Campi R, Pecoraro A, Marzi LV, Tuccio A, Giancane S, Peris A, Cirami CL, Breda A, Vignolini G, Serni S (2022) Robotic Versus Open Kidney Transplantation from Deceased Donors: A Prospective Observational Study. Eur Urol Open Sci 39:36–46. 10.1016/j.euros.2022.03.007 35528789 10.1016/j.euros.2022.03.007 PMC9068739

[2] Zeuschner P, Siemer S, Stöckle M (2020) Roboterassistierte Nierentransplantation. Urologe A 59(1):3–9. 10.1007/s00120-019-01085-9 31832746 10.1007/s00120-019-01085-9

[3] Vignolini G, Campi R, Sessa F, Greco I, Larti A, Giancane S, Sebastianelli A, Gacci M, Peris A, Marzi LV, Breda A, Siena G, Serni S (2019) Development of a robot-assisted kidney transplantation programme from deceased donors in a referral academic centre: technical nuances and preliminary results. BJU Int 123(3):474–484. 10.1111/bju.14588 30311992 10.1111/bju.14588

[4] Prudhomme T, Beauval JB, Lesourd M, Roumiguié M, Decaestecker K, Vignolini G, Campi R, Serni S, Territo A, Gausa L, Tugcu V, Sahin S, Alcaraz A, Musquera M, Stockle M, Janssen M, Fornara P, Mohammed N, Del Bello A, Kamar N, Sallusto F, Breda A, Doumerc N (2021) Robotic-assisted kidney transplantation in obese recipients compared to non-obese recipients: the European experience. World J Urol 39(4):1287–1298. 10.1007/s00345-020-03309-6 32562044 10.1007/s00345-020-03309-6

[5] Oberholzer J, Giulianotti P, Danielson KK, Spaggiari M, Bejarano-Pineda L, Bianco F, Tzvetanov I, Ayloo S, Jeon H, Garcia-Roca R, Thielke J, Tang I, Akkina S, Becker B, Kinzer K, Patel A, Benedetti E (2013) Minimally invasive robotic kidney transplantation for obese patients previously denied access to transplantation. Am J Transplant 13(3):721–728. 10.1111/ajt.12078 23437881 10.1111/ajt.12078 PMC3647345

[6] Doumerc N, Roumiguié M, Rischmann P, Sallusto F (2015) Totally Robotic Approach with Transvaginal Insertion for Kidney Transplantation. Eur Urol 68(6):1103–1104. 10.1016/j.eururo.2015.07.026 26215608 10.1016/j.eururo.2015.07.026

[7] Vignolini G, Greco I, Sessa F, Gemma L, Pecoraro A, Barzaghi P, Grosso A, Corti F, Mormile N, Martiriggiano M, Berni A, Firenzuoli N, Gacci M, Giancane S, Sebastianelli A, Marzi LV, Serni S, Campi R (2020) The University of Florence Technique for Robot-Assisted Kidney Transplantation: 3‑Year Experience. Front Surg 7:583798. 10.3389/fsurg.2020.583798 33262999 10.3389/fsurg.2020.583798 PMC7686135

[8] Yamaoka Y, Yamaguchi T, Kinugasa Y, Shiomi A, Kagawa H, Yamakawa Y, Furutani A, Manabe S, Torii K, Koido K, Mori K (2019) Mesorectal fat area as a useful predictor of the difficulty of robotic-assisted laparoscopic total mesorectal excision for rectal cancer. Surg Endosc 33(2):557–566. 10.1007/s00464-018-6331-9 30006838 10.1007/s00464-018-6331-9

[9] Campi R, Pecoraro A, Piramide F, Gallo ML, Serni S, Mottrie A, Territo A, Decaestecker K, Breda A (2024) The ERUS course on robot-assisted kidney transplantation. World J Urol 42(1):205. 10.1007/s00345-024-04802-y 38554210 10.1007/s00345-024-04802-y PMC10981625

